# Metabolic engineering and classical selection of the methylotrophic thermotolerant yeast *Hansenula polymorpha* for improvement of high-temperature xylose alcoholic fermentation

**DOI:** 10.1186/s12934-014-0122-3

**Published:** 2014-08-20

**Authors:** Olena O Kurylenko, Justyna Ruchala, Orest B Hryniv, Charles A Abbas, Kostyantyn V Dmytruk, Andriy A Sibirny

**Affiliations:** 1 Department of Molecular Genetics and Biotechnology, Institute of Cell Biology, National Academy of Sciences of Ukraine, Drahomanov Street, 14/16, Lviv, 79005 Ukraine; 2 Department of Biotechnology and Microbiology, University of Rzeszow, Zelwerowicza 4, Rzeszow, 35-601 Poland; 3 ADM Research, Decatur, IL 62526 USA

**Keywords:** 3-Bromopyruvate, High-temperature fermentation, Xylose, Fuel ethanol, *Hansenula polymorpha*

## Abstract

**Background:**

The methylotrophic yeast, *Hansenula polymorpha* is an industrially important microorganism, and belongs to the best studied yeast species with well-developed tools for molecular research. The complete genome sequence of the strain NCYC495 of *H. polymorpha* is publicly available. Some of the well-studied strains of *H. polymorpha* are known to ferment glucose, cellobiose and xylose to ethanol at elevated temperature (45 – 50°C) with ethanol yield from xylose significantly lower than that from glucose and cellobiose. Increased yield of ethanol from xylose was demonstrated following directed metabolic changes but, still the final ethanol concentration achieved is well below what is considered feasible for economic recovery by distillation.

**Results:**

In this work, we describe the construction of strains of *H. polymorpha* with increased ethanol production from xylose using an ethanol-non-utilizing strain (2EthOH^−^) as the host. The transformants derived from 2EthOH^−^ overexpressing modified xylose reductase (*XYL1m*) and native xylitol dehydrogenase (*XYL2*) were isolated. These transformants produced 1.5-fold more ethanol from xylose than the original host strain. The additional overexpression of *XYL3* gene coding for xylulokinase, resulted in further 2.3-fold improvement in ethanol production with no measurable xylitol formed during xylose fermentation. The best ethanol producing strain obtained by metabolic engineering approaches was subjected to selection for resistance to the known inhibitor of glycolysis, the anticancer drug 3-bromopyruvate. The best mutant selected had an ethanol yield of 0.3 g/g xylose and produced up to 9.8 g of ethanol/l during xylose alcoholic fermentation at 45°C without correction for ethanol evaporation.

**Conclusions:**

Our results indicate that xylose conversion to ethanol at elevated temperature can be significantly improved in *H. polymorpha* by combining methods of metabolic engineering and classical selection.

## Background

The production of bioethanol from renewable feedstocks represents the largest industrial fermentation process with over 85.2 billion liters of fuel ethanol produced in 2012 [1]. Currently ethanol is produced from traditional feedstocks (corn starch and sugarcane) and is known as 1^st^ generation biofuel. Due to increased production of ethanol and the uses of these feedstocks for food and feed applications, no significant growth in 1^st^ generation ethanol production is possible. This is the main reason for continued interest in the development of cost effective technology for the production of the 2^nd^ generation bioethanol from lignocellulosics. These feedstocks primarily consist of commodity crop processing residues, field residues and wood waste processing residues. Fast growing energy crops such as switch grass, miscanthus and trees that can be cultivated in poor or marginal soils have also been touted as possible sources of lignocellulosic feedstocks. In spite of the potential availability of lower cost lignocellulosic feedstocks, the complex structure of these has hindered the development of a cost effective technologies for 2^nd^ generation bioethanol production. The major problems which prevent large scale bioethanol production from lignocellulosics are the absence of: environmentally friendly and cheap technology for lignocellulose pretreatment and hydrolysis and the unavailability of microbial strains which efficiently ferment the major pentose sugars of hemicellulose, of which xylose is the most abundant sugar [2–4]. Some of the different approaches used to overcome these bottlenecks are the development of Consolidated Bioprocessing (CBP) for the pretreatment, hydrolysis and fermentation of lignocellulose to ethanol and the use of Simultaneous Saccharification and Fermentation (SSF) process to ferment pretreated lignocellulose [5,6]. In the last process, the pretreated lignocellulose is subjected to enzymatic hydrolysis by cellulases and hemicellulases during the fermentation which converts liberated sugars to ethanol and thus avoids end product inhibition of the enzymes by these sugars. During SSF, microorganisms which efficiently ferment pentose and hexose sugars are utilized [7]. As cellulases and hemicellulases express maximal activities at temperatures in the range 50 – 60°C, it is desirable that the microorganisms used for SSF have higher thermotolerance than the currently used industrial ethanologens [8].

There are many promising ethanologenic microorganisms capable of fermenting the major pentose sugar, xylose. The list includes natural xylose-fermenting yeasts, such as *Pichia (Scheffersomyces) stipitis, Candida shehatae, Pachysolen tannophilus*, *Spathaspora passalidarum,* recombinant yeast *Saccharomyces cerevisiae*, and several recombinant ethanologenic bacteria such as *Escherichia coli, Klebsiella oxytoca, Zymomonas mobilis,* several sp of *Bacillus and Lactobacillus* [3,9–12]. Most of these ethanologens are mesophilic organisms that cannot grow and ferment at temperatures above 40–42°C. A number of ethanologenic high temperature anaerobic bacteria such as *Thermoanaerobacterium saccharolyticum* are promising organisms capable of high-temperature xylose fermentation [13]. However, bacteria have some technological disadvantages when compared to yeasts, such as susceptibility to bacteriophage lysis, uncertainty in regulation that hinder the use of their biomass for feed, and some, unlike yeast, have not been in use at industrial scale. With the exception of *Kluyveromyces marxianus* [14], *Hansenula polymorpha*, is the most thermotolerant yeast known with growth up to a maximal temperature of 50°C [15]. It has been demonstrated that *H. polymorpha* can ferment glucose, cellobiose and xylose [16] and is able to convert glycerol to ethanol [17]. Wild-type strains of this yeast normally ferment xylose up to a maximal temperature of 48°C, whereas at 50°C fermentation is strongly suppressed. Genetic manipulation leading to increase in intracellular trehalose following knock out of acid trehalase gene *ATH1* or the overexpression of the heat shock proteins Hsp16 and Hsp104, have been demonstrated to allow normal xylose fermentation at 50°C [18]. The ethanol tolerance of *H. polymorpha* can be further increased by the overexpression of the heterologous gene *MPR1* [19] or endogenous gene *ETT1* [Ishchuk O, Abbas C, Sibirny A, in preparation]. The methylotrophic yeast, *H. polymorpha* is an industrially important microorganism, and belongs to the best studied yeast species with well-developed tools for molecular research [20,21]. The complete genome sequence of the strain NCYC495 of *H. polymorpha* is publicly available [22].

*H. polymorpha* can be a promising organism for both CBP and SSF processes. Recombinant strains of this organism have been engineered which express amylolytic and xylanolytic enzymes and directly ferment starch and xylan to ethanol [23]. Considering SSF process, *H. polymorpha* belongs to very few thermotolerant yeast species capable of xylose fermenting and could be the organism of choice, however, it is not free from several drawbacks. Most importantly, ethanol yield and productivity from xylose in wild-type strains of *H. polymorpha* are very low. However, these features could be improved by classical selection and metabolic engineering. Three approaches have been used earlier for construction of *H. polymorpha* strains which improved ethanol production from xylose. In one line of investigation, *H. polymorpha* gene *XYL1* coding for xylose reductase (XR), two paralogs of xylitol dehydrogenase (XDH) *XYL2A* and *XYL2B,* were deleted in the strain CBS4732 with the expression of the bacterial gene *xylA* from *E. coli* or *Streptomyces coelicolor* [24]. The corresponding transformants expressed xylose isomerase activity and grew on xylose, but the amount of accumulated ethanol was very low (both transformants and wild-type cells accumulated maximally 0.15 g of ethanol/l). The overexpression of *E. coli xylA* together with *H. polymorpha XYL3* coding for xylulokinase (XK), increased ethanol production, however, maximal ethanol accumulation did not exceed 0.6 g/l at 48°C [25]. In the second line of investigation, the *H. polymorpha* XR gene was engineered by site specific mutagenesis to reduce affinity toward NADPH using a similar approach developed for *Candida tenuis* [26]. Consequently, genes coding for modified XR (*XYL1m*), native XDH (*XYL2*) and XK (*XYL3*) were overexpressed in strain CBS4732 which yielded a 2-fold higher ethanol accumulation in corresponding transformants reaching 1.3 g of ethanol/l [27]. In third line of investigation, the wild-type *H. polymorpha* strain NCYC495 (currently, the sequenced strain) was selected as the initial host as it was shown to be a more efficient xylose fermenter relative to the strain CBS4732. The *H. polymorpha* mutant 2EthOH^−^ unable to utilize ethanol as a sole carbon source was isolated from strain NCYC495 and characterized by a 3-fold increase in ethanol accumulation. Subsequently, the gene *PDC1* coding for pyruvate decarboxylase (PDC) was cloned and overexpressed in strain 2EthOH^−^. The best selected transformants from 2EthOH^−^ accumulated 2.5 g of ethanol/l at 48°C [28]. This concentration is still very low and has to be substantially increased before the strain can meet requirements for industrial production using an SSF process.

In the current work, we describe the construction of more efficient *H. polymorpha* high-temperature ethanol producers from xylose. For this, the combination of methods of metabolic engineering and classical selection were applied. Strain 2EtOH^−^ was used for overexpression of *H. polymopha* genes *XYL1m, XYL2, XYL3* and *PDC1*. The best selected transformant was used for the isolation of mutants resistant to the anticancer drug 3-bromopyruvate which is known to inhibit glycolysis [29–31]. The best mutant obtained showed a 15-fold enhancement in ethanol synthesis from xylose when compared to the wild-type strain accumulating up to 10 g/l of ethanol at 45°C.

## Results

### Construction of strains overexpressing engineered XR and native XDH

**Figure 1:**
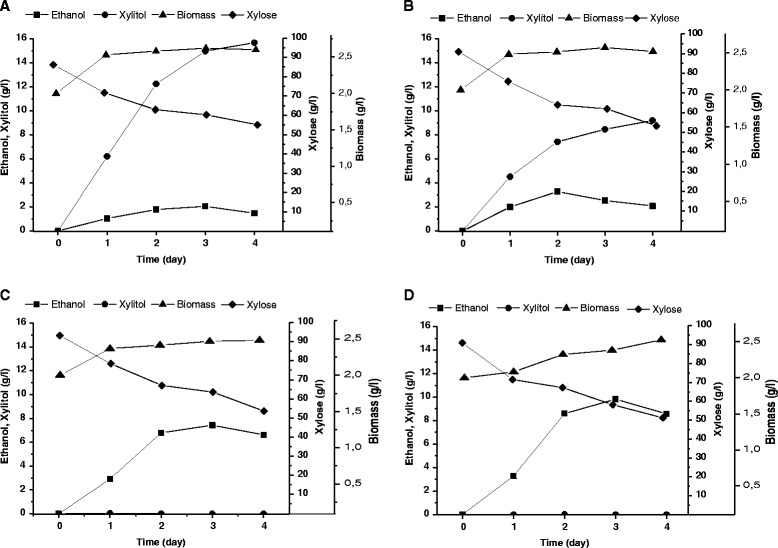
**The ethanol and xylitol production, xylose consumption and biomass accumulation during xylose fermentation at 45°C of** ***H. polymorpha*** **strains.** 2EthOH^−^ **(A)**, 2EthOH^−^/XYL1m/XYL2 **(B)**, 2EthOH^−^/XYL1m/XYL2/XYL3 **(C)**, 2EthOH^−^/XYL1m/XYL2/XYL3/BrPA **(D)**. The data represent values of typical single fermentation.

Analysis of our previous data showed that the highest ethanol yield from xylose was achieved using an ethanol-non-utilizing strain that was derived from strain NCYC495. The corresponding mutant 2EthOH^−^ was characterized by a 3-fold higher ethanol yield on the third day of xylose fermentation, relative to the wild-type strain. The underlying molecular nature of the mutation in the strain 2EthOH^−^ is not known as can be caused by a change in one of the alcohol dehydrogenase isozymes [28]. Recently, it was also shown that overexpression of certain genes in *H. polymorpha* led to an increase in ethanol production from xylose. These changes may result from the cloning of an engineered XR and native XDH, XK and PDC (*XYL1m, XYL2, XYL3, PDC1*) [25,27,28]. We hypothesized that introduction of additional copies of these genes into the genome of the mutant strain 2EthOH^−^ could further improve ethanol production during xylose fermentation. For the co-overexpression of genes *XYL1m* and *XYL2* under control of the strong constitutive promoter of the gene *GAP1* encoding glyceraldehyde-3-phosphate dehydrogenase, the plasmid pX1m-Z-X2 was constructed and used to transform strain 2EthOH^−^. Selection of transformants was performed on media supplemented with increased concentration of the selective agent zeocin assuming multicopy integration of the plasmid bearing the *Zeo*^*R*^ selective marker gene. Transformants were selected on medium with 0.3 g of zeocin/l. After stabilization via cultivation in non-selective medium for 10–12 generations, the cells were grown in a selective medium and subjected to biochemical analyses. The best obtained transformant 2EthOH^−^/XYL1m/XYL2 was characterized by a 1.7-fold increase in specific activity of XR and a 10-fold higher activity of XDH (Table 1). The overexpression of *XYL1m* and *XYL2* genes resulted in a 1.5-fold increase in ethanol production during xylose fermentation in batch culture with limited aeration reaching 3.3 g/l of ethanol at 45°C with a 1.7-fold reduction in xylitol production when compared to the parental strain (Table 2, Figure 1).

**Table 1:** **XR, XDH, XK, PDC activities of** ***H. polymorpha*** **transformants and control strain**

**Strain**	**Activity (U/mg protein)**			
	**XR**	**XDH**	**XK**	**PDC**
2EthOH^−^	0.041 ± 0.002	0.11 ± 0.005	0.10 ± 0.005	0.26 ± 0.013
2EthOH^−^/XYL1m/XYL2	0.069 ± 0.003	1.12 ± 0.052	0.12 ± 0.006	0.32 ± 0.011
2EthOH^−^/XYL1m/XYL2/XYL3	0.075 ± 0.004	1.15 ± 0.057	0.31 ± 0.012	-
2EthOH^−^/XYL1m/XYL2/PDC1	0.068 ± 0.003	1.09 ± 0.054	-	5.38 ± 0.091
2EthOH^−^/XYL1m/XYL2/XYL3/PDC1	0.073 ± 0.004	1.08 ± 0.053	0.21 ± 0.009	2.42 ± 0.089
2EthOH^−^/XYL1m/XYL2/XYL3/BrPA	0.072 ± 0.004	1.21 ± 0.061	0.33 ± 0.015	-

-not determined.

**Table 2:** **Ethanol productivity, ethanol and xylitol yield of** ***H. polymorpha*** **transformants and control strain**

**Strain**	**Ethanol (g/l)**	**Ethanol yield (g/g consumed xylose)**	**Ethanol specific production rate (g/g biomass/h)**	**Ethanol productivity (g/l/h)**	**Xylitol (g/l)**	**Xylitol yield (g/g consumed xylose)**
2EthOH^−^	2.054 ± 0.103	0.080 ± 0.004	0.017 ± 0.001	0.043 ± 0.002	15.657 ± 0.078	0.6074 ± 0.035
2EthOH^−^/XYL1m/XYL2	3.285 ± 0.164	0.113 ± 0.007	0.034 ± 0.002	0.083 ± 0.004	8.458 ± 0.042	0.2912 ± 0.019
2EthOH^−^/XYL1m/XYL2/XYL3	7.441 ± 0.371	0.253 ± 0.031	0.059 ± 0.003	0.142 ± 0.007	0	0
2EthOH^−^/XYL1m/XYL2/PDC1	4.732 ± 0.235	0.121 ± 0.005	0.040 ± 0.002	0.094 ± 0.005	6.751 ± 0.031	0.241 ± 0.015
2EthOH^−^/XYL1m/XYL2/XYL3/PDC1	5.043 ± 0.249	0.163 ± 0.009	0.042 ± 0.002	0.105 ± 0.005	1.104 ± 0.012	0.036 ± 0.005
2EthOH^−^/XYL1m/XYL2/XYL3/BrPA	9.817 ± 0.411	0.300 ± 0.011	0.077 ± 0.004	0.180 ± 0.009	0	0

The data are based on 7 independent replicate cultivations and ± values represent standard deviations.

### Selection of strains co-expressing XR and XDH in part with XK and/or PDC

For further improvement of ethanol production from xylose the genes *XYL3* or *PDC1* were separately or in combination overexpressed in the background of the former strain 2EthOH^−^/XYL1m/XYL2 using the expression vectors pGLG61/XYL3, pGLG61 + prGAP + PDC1*Hp* and pGLG61/XYL3/PDC1. The abovementioned plasmids were derived of pGLG61 and contain a weakly expressed bacterial gene *APH* encoding aminoglucoside-3-phosphotransferase as the dominant marker for geneticin (G418) resistance, and the sequence of the *HARS36* (*TEL188*) as autonomic replicating sequence. Such elements ensure multiple tandem integration of pGLG61 into the *H. polymorpha* telomere regions on a medium containing geneticin [32]. Transformants expressing *XYL3* and both *XYL3* and *PDC1* genes were selected on a medium supplemented with 0.5 g/l geneticin while the strains expressing *PDC1* were selected on a medium with up to 1.0 g/l of this antibiotic. All types of transformants were stabilized. The presence of the corresponding plasmids in the stabilized transformants was confirmed by diagnostic PCR.

The expression of *XYL3* gene in the background of 2EthOH^−^/XYL1m/XYL2 resulted in up to 2.6-fold increase in the specific activity of XK in selected transformant 2EthOH^−^/XYL1m/XYL2/XYL3 when compared to the parental strain. The recombinant strain 2EthOH^−^/XYL1m/XYL2/XYL3/PDC1 co-expressing *XYL3* and *PDC1* genes was shown to have 1.8- and 7.6-fold enhancement of XK and PDC activity when compared to the initial strain. The specific activity of the enzyme PDC in strain 2EthOH^−^/XYL1m/XYL2/PDC1 was 18-fold increased when compared to strain 2EthOH^−^/XYL1m/XYL2 (Table 1). Such remarkable increase in PDC activity can only be explained by the multicopy integration of the plasmid pGLG61 + prGAP + PDC1*Hp* into the engineered strain 2EthOH^−^/XYL1m/XYL2/PDC1 obtained following selection on the medium supplemented with 1 g of geneticin/l. All other obtained strains harboring additional copies of *XYL3* or/and *PDC1* gene, were characterized by identical specific activity of XR and XDH (Table 1). Results of ethanol production at the third day of xylose fermentation at 45°C by the constructed strains are shown in Table 2. The highest ethanol production was observed for strain 2EthOH^−^/XYL1m/XYL2/XYL3. Such a strain showed a 2.3-fold improvement in ethanol production relative to the parental strain 2EthOH^−^/XYL1m/XYL2. Strains 2EthOH^−^/XYL1m/XYL2/PDC1 and 2EthOH^−^/XYL1m/XYL2/XYL3/PDC1, synthesized approximately 1.5-fold higher amounts of ethanol than that of parental strain. On the 3^rd^ day of xylose fermentation, the best selected strains expressing singly *XYL3, PDC1* or a combination of these two genes in the background of strain 2EthOH^−^/XYL1m/XYL2, accumulated 7.44 g/l, 4.7 g/l and 5.0 g/l of ethanol, respectively (Table 2). Thus, the expression of the *PDC1* gene in the strain co-expressing all three genes *XYL1m, XYL2, XYL3* involved in the initial stages of xylose catabolism had no positive effect on ethanol production, though overexpression of *PDC1* alone in the background of 2EthOH^−^/XYL1m/XYL2 strain is beneficial for ethanol synthesis. The impact of *XYL3* gene on ethanol production during xylose fermentation is more significant as compared to *PDC1* gene, as overexpression of the *XYL3* gene resulted in reduced xylitol accumulation during alcoholic fermentation of xylose. The strain 2EthOH^−^/XYL1m/XYL2 produced 8.5 g of xylitol /l with xylitol yield of 0.29 g/g from consumed xylose (Table 2, Figure 1). Expression of gene *XYL3* resulted in a significant reduction of xylitol synthesis to zero. By comparison, the overexpression of *PDC1* had no influence on xylitol accumulation. The expression of *PDC1* in combination with *XYL3,* reduced xylitol yield to 0.04 g/g caused by an increased activity of XK (Table 1). The difference between xylitol production of strains 2EthOH^−^/XYL1m/XYL2/XYL3 and 2EthOH^−^/XYL1m/XYL2/XYL3/PDC1 can be explained by the alteration in the specific activity of this enzyme. The activity of XK of strain 2EthOH^−^/XYL1m/XYL2/XYL3 was 1.5-fold higher than that of strain 2EthOH^−^/XYL1m/XYL2/XYL3/PDC1 and this correlated with an increase in copy number of *XYL3* gene in the genomes of both strains (Table 1). This demonstates that sufficient activity of XK is necessary for the reduction of xylitol formation during xylose alcoholic fermentation. If the level of XK is not high enough for complete direction of xylulose to xylulose-5-phosphate, xylulose can be reduced back to xylitol via the reverse reaction catalyzed by XDH [33,34].

Thus, the overexpression of three enzymes involved in the initial stages of xylose catabolism, modified XR, native XDH and XK, in the background of an ethanol-non-utilizing mutant 2EthOH^−^, significantly improved ethanol yield from xylose (0.25 g/g xylose relative to 0.08 g/g xylose in the mutant 2EthOH^−^ after 3 days of xylose fermentation at 45°C), ethanol specific production rate (0.059 g/g biomass/h versus 0.017 g/g biomass/h for parental strain) and ethanol productivity (0.14 g/l/h versus 0.04 g/l/h for initial strain). In spite of the improvements made to xylose alcoholic fermentation, these are not adequate for a profitable SSF process. Additional production of ethanol from xylose was attempted using the best strain isolated via metabolic engineering approaches and subjected to further strain selection by classical approaches.

### Isolation and characterization of the ethanol overproducing strains resistant to anticancer drug 3-bromopyruvate

3-Bromopyruvate (3-BrPA) has been known for some time as a promising anticancer drug. It is well documented that this compound causes ATP depletion which induce cell death as a result of blocking of glycolysis by the inhibition of the glycolytic enzymes, hexokinase II, glyceraldehyde-3-phosphate dehydrogenase and 3-phosphoglycerate kinase. In addition to glycolytic enzymes, 3-BrPA has been shown to have other targets of action [29]. We hypothesized that yeast mutants resistant to 3-BrPA can have mutation in regulatory or structural glycolytic genes leading to increased glycolytic flux and as a result elevated amount of synthesized ethanol during fermentation. In our experiments, the best ethanol-producing strain 2EthOH^−^/XYL1m/XYL2/XYL3 co-overexpressing *XYL1m, XYL2* and *XYL3* genes, was used as target for further selection and improvement. Mutants resistant to 3-BrPA were isolated on solid mineral medium containing xylose as the sole carbon source and supplemented with 0.11 mM 3-BrPA. Around 150 mutants resistant to 3-BrPA were selected and evaluated for the ethanol production during xylose fermentation. The 110 selected mutants possessed increased ethanol production as compared to the parental strain. In other words, approximately 70% of 3-BrPA-resistant mutants isolated displayed increase in ethanol synthesis during xylose fermentation. The average ethanol yield was increased on 10-20% as compared to the parental strain. Biochemical characteristics and ethanol production of the best selected mutant 2EthOH^−^/XYL1m/XYL2/XYL3/BrPA is presented in Table 1. The 2EthOH^−^/XYL1m/XYL2/XYL3/BrPA mutant was characterized by approximately 1.3-fold improvement in the ethanol yield as compared to 2EthOH^−^/XYL1m/XYL2/XYL3 strain reaching 0.3 g/g xylose. This 3-BrPA-resistant mutant had no measurable changes in the specific activities of XR, XDH and XK, as well as in xylitol production relative to the parental strain 2EthOH^−^/XYL1m/XYL2/XYL3 (Table 1).

Representative profiles of ethanol synthesis, biomass accumulation, xylose consumption and xylitol formation for the strains 2EthOH^−^, 2EthOH^−^/XYL1m/XYL2, 2EthOH^−^/XYL1m/XYL2/XYL3 and 2EthOH^−^/XYL1m/XYL2/XYL3/BrPA are shown in Figure 1. The consumption of xylose by constructed strains as well as by the initial parental 2EthOH^−^ strain during fermentation was incomplete, suggesting that xylose uptake could be a serious bottleneck leading to inefficient xylose conversion to ethanol and this presents another potential target for further improvement of ethanol production from xylose.

The best obtained 2EthOH^−^/XYL1m/XYL2/XYL3/BrPA mutant accumulated 9.8 g/l of ethanol after 3 days of fermentation. The synthesized ethanol concentration was corrected for evaporation at high temperature xylose fermentation experiments. It was found that in model experiments when ethanol at fixed concentrations was shaken in fermentation medium under fermentation conditions, approximately 50% of it was evaporated at 45°C in 3 days of incubation (data not shown). Correction for ethanol evaporation gives the calculated amounts of accumulated ethanol as approximately 20 g/l on the 3^rd^ day of xylose fermentation at 45°C. The strain 2EthOH^−^/XYL1m/XYL2/XYL3/BrPA, similarly to the parental strain 2EthOH^−^/XYL1m/XYL2/XYL3, did not accumulate xylitol in the medium.

## Discussion

*H. polymorpha* belongs to the best studied yeast species with well-developed tools for molecular research. It is used commercially for production of recombinant vaccines, interferons, insulin, enzymes and other products [21,22]. Its ability to ferment xylose to ethanol at elevated temperature is known for 10 years; however, ethanol yield and productivity are very low. At the same time, it rather efficiently converts glucose, cellobiose and glycerol to ethanol [17,18]. Several metabolic engineering approaches have been successfully developed to improve ethanol production from xylose in *H. polymorpha*, however, ethanol production remained quite low [24,25,27,28]. In the current work, we decided to combine several developed earlier approaches of metabolic engineering with classical selection for ethanol overproducing strain using selection for antimetabolite resistance. We demonstrated that there is a positive cumulative effect for the overexpression of engineered XR and native XDH and XK on ethanol production from xylose. Additional overexpression of *PDC1* gene coding for PDC did not lead to further improvement of ethanol synthesis from xylose, though overexpression of *PDC1* in the background of *XYL1m* and *XYL2* overexpressed strain increased ethanol production. The impact of XK on ethanol production during xylose alcoholic fermentation is more pronounced when compared to PDC assuming that PDC does not limit xylose conversion in strain expressing XR, XDH and XK. Overexpression of *XYL1m, XYL2* and *XYL3* in the background of non-identified mutation in the strain 2EthOH^−^, led to substantial increase in ethanol accumulation during xylose fermentation (7.44 g/l at 45°C relative to 0.6 g/l in the wild-type strain NCYC495 and 2.05 g/l in the parental strain 2EthOH^−^).

The method we used for selection of the ethanol-overproducers among 3-BrPA-resistant mutants has been, to our knowledge, demonstrated for the first time to increase ethanol production in yeasts. It is interesting to note that 70% of 3-BrPA resistant mutants obtained in this work, produced more ethanol as compared to the parental strain in xylose medium. High percentage of ethanol overproducers provides support for the use of this method for positive selection of ethanol overproducers. In mammalian cells 3-BrPA has multiple targets of action [29–31]. It would be important to identify targets of action of this inhibitor in yeast cells and gene(s) which mutations lead to 3-BrPA resistance and ethanol overproduction. We also have found that selection of 3-BrPA-resistant mutants can be successfully used for the isolation of ethanol-overproducing strains in other yeast species, including *S. cerevisiae* (unpublished observation). The exact molecular events underlying 3-BrPA resistance in mutants of *S. cerevisiae* and *H. polymorpha* will be described from another separate study [Hryniv, O., Dmytruk, K., Sibirny, A., in preparation].

Thus, in this work we were able to increase accumulation of ethanol from xylose to 10 g/l relative to 0.6 g/l in the wild-type strain, or more than 15 times by combining the approaches of metabolic engineering and classical selection. The maximal observed level of ethanol produced from xylose by the best isolated strains (near 10 g/l at 45°C results in an ethanol yield of 0.3 g/g from xylose). These results make *H. polymorpha* close to known promising organisms for the use in SSF process. Still this ethanol concentration is lower than ethanol production in mesophilic xylose fermenting organisms such as *P. stipitis* (0.35-0.44 g/g xylose) and *S. passalidarum* (0.42 g/g xylose), but similar to the best engineered strain of thermotolerant yeast *K. marxianus* (0.31 g/g xylose under anaerobic conditions at 45°C) [12,35,36]. However, the ethanol productivity in the best *H. polymorpha* isolated strain is much higher when compared to the best engineered strain of *K. marxianus* (0.179 g/l/h versus 0.054 g/l/h at 45°C). To be industrially feasible, ethanol yield in *H. polymorpha* has to be further increased to be close to the theoretical maximum. We are currently using other targets for metabolic engineering (e.g. xylose transport, pentose phosphate pathway). This we hope can lead to further increase in ethanol yield from xylose during high-temperature alcoholic fermentation of this promising organism.

## Conclusions

Methods of metabolic engineering and classical selection were successfully applied for construction of more efficient *H. polymorpha* ethanol producers from xylose, leading to 15-fold enhancement in ethanol synthesis from xylose as compared to the wild-type strain.

## Materials and methods

### Strains, media, cultivation conditions

Yeast strain *H. polymorpha* 2EthOH^−^, a UV-induced mutant derived from the parental strain NCYC495 *leu1-1*, and transformants listed in Table 3 were grown on YPD (10 g/l yeast extract, 10 g/l peptone, 20 g/l glucose) or mineral medium (6.7 g/l YNB without amino acids, 40 g/l xylose or 20 g/l glucose) at 37°C. For the 2EthOH^−^ strain, leucine (40 mg/l) was added to the medium. For the selection of yeast transformants on YPD, 0.15 – 0.3 g/l of zeocin or 0.5 – 1.0 g/l of geneticin were added. For the isolation of 3-bromopyruvate resistant mutants, a mineral medium containing glucose or xylose as sole carbon sources with 0.05-0.11 mM of selective agent was used.

**Table 3:** **Yeast strains and plasmids used in this study**

**Strains**	**Genotype**	**References**
2EthOH^−^	*leu2*	Ishchuk et al., 2008 [28]
2EthOH^−^/XYL1m/XYL2	pX1M-Z-X2 *(GAPp-XYL1mod-AOXt, GAPp-XYL2-XYL2t)*	This study
2EthOH^−^/XYL1m/XYL2/XYL3	pX1M-Z-X2 *(GAPp-XYL1mod-AOXt, GAPp-XYL2-XYL2t)*, pGLG61/XYL3 (*GAPp-XYL3-AOXt*)	This study
2EthOH^−^/XYL1m/XYL2/XYL3/BrPA	As above with unfixed mutations caused by selection on 3-BrPA containing medium	This study
2EthOH^−^/XYL1m/XYL2/PDC1	pX1M-Z-X2 *(GAPp-XYL1mod-AOXt, GAPp-XYL2-XYL2t)*, pGLG61 + prGAP + PDC1Hp (*GAPp-PDC1-AOXt*)	This study
2EthOH^−^/XYL1m/XYL2/XYL3/PDC1	pX1M-Z-X2 *(GAPp-XYL1mod-AOXt, GAPp-XYL2-XYL2t)*, pGLG61/XYL3/PDC1 (*GAPp-XYL3-AOXt*, *GAPp-PDC1-AOXt*)	This study

The *E. coli* DH5α strain (Φ80d*lacZ*ΔM15, *recA*1, *endA*1, *gyrA*96, *thi*-1, *hsdR*17(r_K_^−^, m_K_^+^), *supE*44, *relA*1, *deoR*, Δ(*lacZYA-argF*)U169) was used as a host for plasmid propagation. Strain DH5α was grown at 37°C in LB medium as described previously [37]. Transformed *E. coli* cells were maintained on a medium containing 100 mg/l of ampicillin.

### Molecular-biology techniques

Standard cloning techniques were used as described [37]. Genomic DNA of *H. polymorpha* was isolated using the Wizard® Genomic DNA Purification Kit (Promega, Madison, WI, USA). Restriction endonucleases and DNA ligase (Fermentas, Vilnius, Lithuania) were used according to the manufacturer specifications. Plasmid isolation from *E. coli* was performed with the Wizard® *Plus* SV Minipreps DNA Purification System (Promega, Madison, WI, USA). DNA fragments were separated on a 0.8% agarose (Fisher Scientific, Fair Lawn, NJ, USA) gel. Isolation of fragments from the gel was carried out with a DNA Gel Extraction Kit (Millipore, Bedford, MA, USA). PCR-amplification of the fragments of interest was done with Platinum® *Taq* DNA Polymerase High Fidelity (Invitrogen, Carlsbad, CA, USA) according to the manufacturer specification. PCRs were performed in GeneAmp® PCR System 9700 thermocycler (Applied Biosystems, Foster City, CA, USA). Transformation of the yeast *H. polymorpha* by electroporation was carried out as described previously [38].

### Plasmid construction

**Figure 2:**
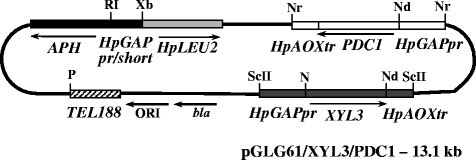
**Scheme of the plasmid pGLG61/XYL3/PDC1.** Expression cassettes *GAPp-XYL3-AOXt* and *GAPp-PDC1-AOXt* are shown as gray and white boxes, respectively. The geneticin resistance gene (*APH*), linked to the impaired constitutive gene promoter, encoding glyceraldehyde-3-phosphate dehydrogenase (*HpGAPpr*) and *H. polymorpha LEU2* gene are shown as black and light-gray boxes, respectively. The telomeric region (TEL188) as an autonomously replicating sequence is designated with the hatched lines. Origin of replication (ORI) and ampicillin resistance gene (*bla*) – arrows. Restriction sites: RI, *Eco*RI; Xb, *Xba*I; ScII, *Sac*II; Nr, *Nar*I; Nd, *Nde*I; N, *Not*I; P, *Pst*I.

Plasmid pX1m-Z-X2 was used for overexpression of modified *XYL1m* gene and native *XYL*2 gene [27]. Plasmids pGLG61/HpXYL3 [25] and pGLG61 + prGAP + PDC1*Hp* [26] were used for overexpression of *XYL3* and *PDC1* genes, respectively*.* For simultaneous overexpression of *XYL3* and *PDC1* genes a NarI-restriction fragment containing *GAPp-PDC1-AOXt* was isolated from the plasmid pGLG61 + prGAP + PDC1*Hp* and cloned into the NarI-linearized and dephosphorylated vector pGLG61/HpXYL3. The resulting plasmid was named pGLG61/XYL3/PDC1 (Figure 2).

### Biochemical methods

The specific activities of XR, XDH and XK in cell extracts were determined spectrophotometrically as described before [27].

The PDC activity in cell extracts was determined spectrophotometrically according to the methods described earlier [39]. Samples for enzyme activity measurements were taken from the cultures on the third day of xylose fermentation at 45°C. The enzyme activity was measured directly after the preparation of cell-free extracts as described before [28].

All assay experiments were repeated at least twice.

### Selection of 3-bromopyruvate (3-BrPA) resistant mutants

For isolation of 3-BrPA-resistant mutants solid mineral medium containing glucose or xylose as sole carbon sources with 0.11 mM of selective agent was used. Cell suspension of 2EthOH^−^/XYL1m/XYL2/XYL3 strain was plated on the 3-BrPA containing medium for final OD_600_ = 0.1/ml and incubated at 37°C for five days. Single 3-BrPA-resistant colonies were picked up, re-streaked on fresh YNB plates containing 0.11 mM of 3-BrPA and used for fermentation experiments. Selected mutants were stable and possessed resistance to the selective agent even after 6 month growth on agar slant cultures in YPD medium with regular transfer to fresh YPD medium every month.

### Analyses

Cells of transformants were grown in 100 ml of YPX medium (10 g/l yeast extract, 20 g/l peptone, 40 g/l xylose) in Erlenmeyer flasks (bottle size - 300 ml) for 2 days and then inoculated into the 40 ml of YNB medium with 90 g/l xylose in 100 ml Erlenmeyer flasks. Fermentation was carried out at a temperature of 45°C with limited aeration (140 revolutions/min). Concentrations of xylose, xylitol and ethanol from fermentation in medium broth were analyzed by HPLC (PerkinElmer, Series 2000, USA) with an Aminex HPX-87H ion-exchange column (Bio-Rad, Hercules, USA). A mobile phase of 4 mM H_2_SO_4_ was used at a flow rate 0.6 ml/min and the column temperature was 35°C. Alternatively concentrations of ethanol in the medium were determined using alcohol oxidase/peroxidase-based enzymatic kit “Alcotest” [40]. For high temperature xylose fermentation experiments, the ethanol concentration was corrected for evaporation. The evaporation coefficient was calculated as a decrease of known concentration of ethanol at 45°C temperature.

Experiments were performed at least twice.

## Abbreviations

SSF: Simultaneous saccharification and Fermentation
CBP: Consolidated bioprocessing
XR: Xylose reductase
XDH: Xylitol dehydrogenase
XK: Xylulokinase
PDC: Pyruvate decarboxylase
3-BrPA: 3-Bromopyruvate
